# A context-aware interpretive framework for lymphocyte immunophenotyping by flow cytometry

**DOI:** 10.3389/fimmu.2026.1788361

**Published:** 2026-05-29

**Authors:** Carlos Agustin Villegas-Valverde, Yendry Ventura-Carmenate

**Affiliations:** 1 Abu Dhabi Stem Cells Center, Abu Dhabi, United Arab Emirates; 2 Abu Dhabi Stem Cells Center and the United Arab Emirates University-College of Medicine, Abu Dhabi, United Arab Emirates

**Keywords:** analytical reproducibility, clinical immunology, flow cytometry, interpretive framework, lymphocyte immunophenotyping

## Abstract

**Background:**

Flow cytometry–based immunophenotyping is a powerful tool in clinical and translational immunology; however, the interpretation of lymphocyte immunophenotype results remains incomplete, despite extensive methodological standardization. This challenge is particularly evident when phenotypically normal lymphocytes are quantified under non-physiological conditions, where numerical results are often interpreted using implicit and heterogeneous assumptions.

**Hypothesis:**

It is hypothesized that harmonization in flow cytometry–based lymphocyte immunophenotyping must extend beyond technical standardization and incorporate explicit, structured frameworks for biological and clinical interpretation for enumeration of phenotypically normal lymphocyte subsets.

**Implications:**

Five foundational guidelines are proposed for interpreting lymphocyte immunophenotypic enumeration: the non-equivalence of relative and absolute metrics, context-dependent relevance of immunophenotypic values, compartment-specific determination of meaning, analytical constraints imposed by assay architecture, and the impact of analytical variability on interpretative stability. By articulating principles that are already implicitly applied in expert practice, this framework complements existing technical guidelines and supports a more consistent, context-aware interpretation of lymphocyte immunophenotypes across diverse clinical and biological settings.

**Conclusion:**

By explicitly articulating principles that are typically implicit in expert practice, this framework complements existing technical guidelines and bridges the gap between technical and interpretative reproducibility. It provides a coherent structure for context-aware immunophenotypic interpretation and contributes to a shared conceptual language for the study of immunology and its clinical application.

## 1 Introduction

Flow cytometry (FC) is one of the most versatile and widely used analytical tools in contemporary immunology research and clinical practice. Despite technical advances in standardizing immunophenotyping of normal lymphocytes, it remains critically incomplete when interpretive inference is treated as an implicit, operator-dependent step rather than an explicit, harmonized framework, linking FC data to biological or clinical inferences. Therefore, similar cytometric outputs can lead to divergent biological conclusions across studies, laboratories, and clinical contexts, limiting true comparability and cross-study validity. In contrast, immunophenotyping of malignant hematologic diseases has seen considerable advances in interpretive guidelines, enabling standardized approaches to this heterogeneous group. This has facilitated the identification of new diseases and improved the accuracy of diagnosis and minimal residual disease assessment (1–5).

In routine practice, lymphocyte immunophenotype counts are frequently interpreted based on implicit assumptions about metric equivalence, some validity of inter-compartmental extrapolations, and the neutrality of analytical frameworks. These assumptions, though rarely made explicit, significantly influence final reports. This limitation is particularly pronounced when immunophenotyping is applied to phenotypically normal lymphocytes in non-physiological contexts. For instance, in various clinical scenarios, including immunodeficiencies, autoimmunity, non-hematological cancers, chronic infections, inflammatory states, and hematopoietic cell transplantation, the primary objective is to evaluate the non-malignant immune compartment as an indicator of immunological status, functional reserve, or adaptive response, rather than characterize malignant cells (6–9). Additionally, immunophenotyping normal and reactive lymphocytes is increasingly essential for diagnosing and monitoring malignant hematologic diseases and for assessing the tumor microenvironment (2, 10–12).

Interpreting lymphocyte counts outside of baseline conditions presents conceptual challenges distinct from those encountered in the diagnosis of malignant hemopathies. These challenges cannot be addressed by paradigms designed to detect clonal aberrancies (13). A further complication is the absence of universal reference intervals. Studies have demonstrated that lymphocyte populations exhibit significant variability related to age, sex, geographic origin, ethnicity, and other biological and environmental factors, necessitating the use of reference ranges rather than fixed cut-off points (6, 14–19). In this context, values that appear low or high may be physiological, adaptive, or clinically relevant, depending on the overall immunological status, underlying condition, and the interpretative framework employed. While experts acknowledge this flexibility, it lacks the conceptual and operational systematization required for consistent application and comparison across studies.

Beyond biological complexity, analytical and technical factors also play a significant role. Decisions regarding the use of relative versus absolute metrics, single or dual-platform counting methods, FC or mass cytometry, as well as panel design, gating strategies, and instrument configuration, directly influence the types of inferences that can be drawn. Additionally, cumulative analytical variability and sources of error can introduce systematic interpretive deviations that, if unrecognized, may be mistaken for genuine biological patterns (2, 20). Efforts to standardize and harmonize the complex field of FC do not fully address the methodology for interpreting results of lymphocyte population quantification (21–25).

This proposed set of guidelines aims to harmonize the interpretation of quantitative immunophenotypic results for non-pathological lymphocyte populations. This would facilitate interlaboratory reproducibility and cross-disciplinary collaboration among specialists and researchers using this technology. These guidelines do not replace existing diagnostic frameworks or technical guidelines but rather complement them by structuring immunological inferences explicitly and coherently.

## 2 Hypothesis and conceptual objective of the work

Harmonization in FC–based immunophenotyping of normal lymphocyte analysis remains incomplete when confined to technical and analytical standardization alone, in which technically reproducible data lead to heterogeneous inferences driven by implicit, expert experience-based interpretive assumptions. Proper harmonization requires the parallel development of structured, explicit, and context-aware interpretive frameworks for immunophenotypic findings. Consequently, we propose that formalizing inductively derived interpretive guidelines from empirical practice and the existing literature can transform immunophenotypic heterogeneity from a descriptive limitation into an interpretive dimension structured, reproducible, and conceptually transferable across studies and clinical settings.

## 3 Immunophenotypic interpretation guidelines for the normal lymphocyte compartment

**Figure 1:**
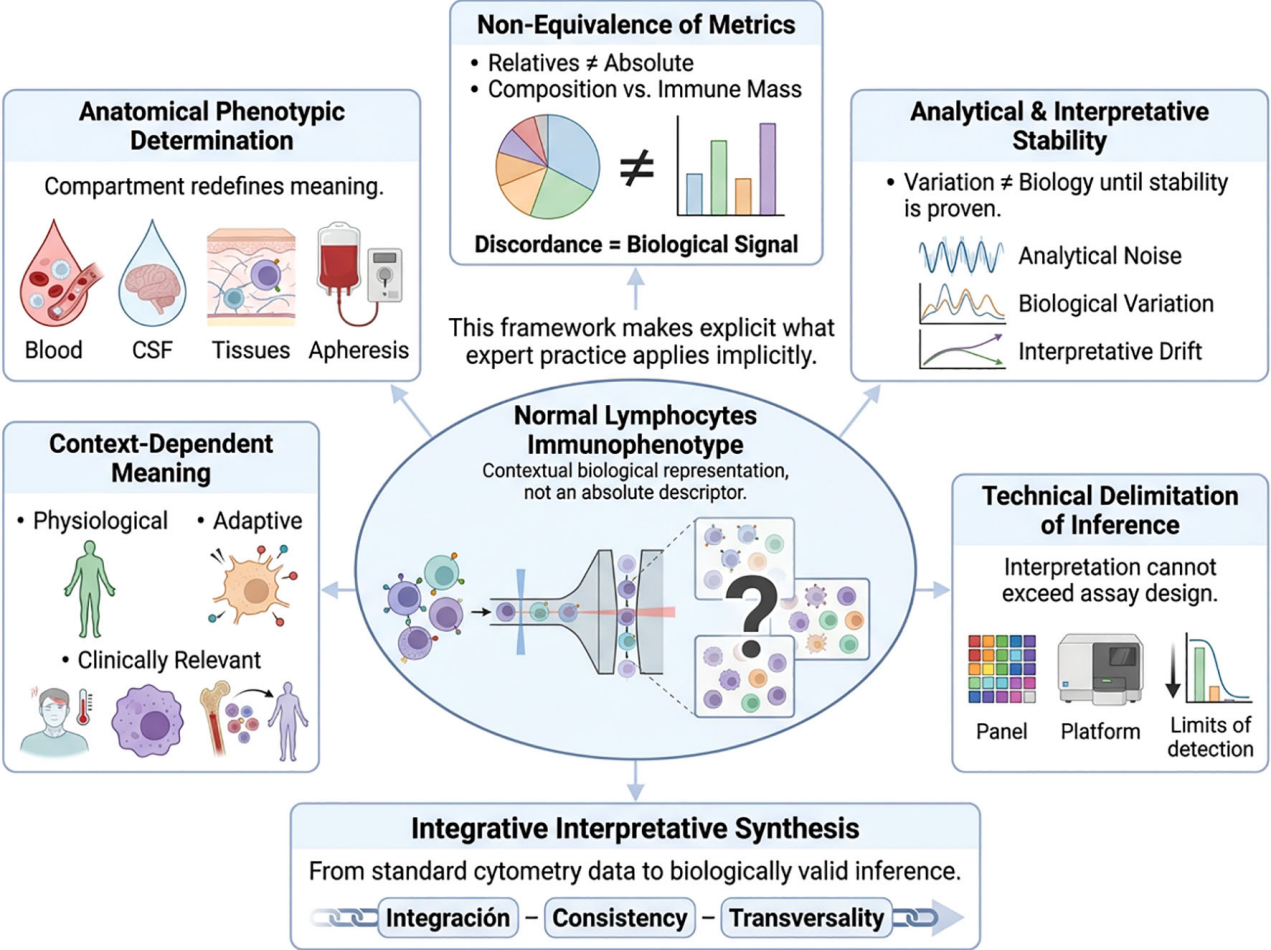
Integrative framework for harmonized interpretation of the normal lymphocyte immunophenotype. This conceptual diagram summarizes the five interpretative guidelines proposed to complement technical standardization in flow cytometry-based immunophenotyping of normal lymphocytes. These include the non-equivalence of relative and absolute metrics, the context-dependent meaning of immunophenotypic values, anatomo-phenotypic determination across biological compartments, technical delimitation of inference imposed by assay architecture, and analytical and interpretative stability required to distinguish biological variation from analytical noise. The central element emphasizes that the normal lymphocyte immunophenotype represents a contextual biological description rather than an absolute descriptor. The lower integrative module illustrates how the convergence of these guidelines enables an integrative interpretative synthesis, transforming standard cytometric data into biologically valid, consistent, and transversal immunological inference, thereby supporting interpretative reproducibility across clinical and translational settings.

The interpretation of the lymphocyte immunophenotype cannot be reduced to a single reading of numerical values or to the automatic application of reference ranges. In real-world clinical and translational immunology practice, the meaning of an immunophenotype emerges from the interaction between metrics, biological context, compartment analyzed, and the technical architecture of the assay. Through the inductive integration of reference studies, analyses of disease, evaluation of unconventional compartments, and analytical standardization processes, a limited set of recurrent interpretive guidelines can be identified that consistently structure inference of immunophenotypic findings in the normal lymphocyte compartment (**Figure 1**).

The guidelines proposed here explicitly draw upon previously established recommendations, many of which are scattered across technical documents, analytical standards, and disease-specific guidelines. Rather than replacing them, this framework’s novelty lies in integrating and harmonizing them within an interpretive structure geared toward the clinical reading of lymphocyte immunophenotyping results. Consequently, its primary emphasis is not on how cytometric data are generated or analyzed, but rather on how the findings should be interpreted once their analytical validity has been established. These guidelines do not constitute diagnostic criteria or closed algorithms but rather reading principles that make explicit the assumptions that are usually implicit in expert interpretations. Their aim was to reduce conceptual ambiguity, facilitate comparability between studies, and improve the robustness of the conclusions derived from cytometric analysis.

### 3.1 Guideline on the non-equivalence of metrics (relative vs. absolute)

Relative metrics (percentages) and absolute metrics (cell concentrations per volume; typically cells/μL) capture fundamentally different biological dimensions of the immune system and are not semantically interchangeable. Percentages describe the internal composition of the lymphocyte compartment and are intrinsically dependent on the denominator (in this case, the reference population is important in the gate strategy), whereas absolute counts reflect the available cell mass.

The flow cytometer generates metrics based on its configuration and the protocol designed by the operator. For absolute counts, there are two conventional methods: single-platform or dual-platform, with the consensus being that single-platform counting is more precise and accurate, whether assisted by counting beads or by volumetry.

The choice of metric is not a detailed report, but an interpretive decision that defines the biological phenomenon being measured. In settings where the total lymphocyte pool remains stable, relative metrics are useful for identifying selective redistribution, polarization, or expansion. It is also useful when you want to measure the purity of a population using a gating strategy. However, under conditions of lymphocyte depletion, immunosuppression, or immune reconstitution, an apparently preserved proportion may coexist with a profound reduction in the absolute lymphocyte count. In such scenarios, relying solely on proportional metrics may mask the biologically relevant immune deficits.

In well-mixed compartments, such as the peripheral blood, relative metrics provide a reliable description of lymphocyte composition when the total lymphocyte pool is stable. However, as the size of the lymphocyte pool becomes variable or reduced, absolute counts take precedence for interpretation, as proportional values ​​can be preserved despite a critical loss of cell mass.

In biological fluids, such as ascites, cerebrospinal fluid, or serous effusions, relative metrics can only accurately reflect compositional balance if adequate cellularity and volume-based normalization are ensured. In samples with low cellularity, the percentages alone can overestimate biological relevance unless accompanied by cell density or absolute counts per volume.

In solid tissues, where lymphocyte distribution is spatially heterogeneous and dominated by localized cell aggregates, neither local relative nor absolute metrics can be directly extrapolated to the entire tissue area. In this context, immunophenotypic values ​​should be interpreted as site-specific descriptors rather than as global representations of the immune compartment. In these cases, cytometric analysis is necessary to complement histological analysis, as has been the case in immunohistochemical studies of mucosa-associated lymphoid tissue. The subphenotype of a population becomes more relevant, as in areas rich in T or B lymphocytes, it is useful to know the composition of the subphenotypes of interest, whether naïve, activated, memory, or senescent.

It is important to emphasize that the discrepancy between relative and absolute values should not be interpreted as a technical artifact (if executed correctly), but rather as an informative signal reflecting a distinct underlying dynamic of the immune compartment and, therefore, requiring an explicit contextual interpretation. To facilitate practical implementation, the non-equivalence of relative and absolute metrics can be translated into compartment-specific interpretative rules, as summarized in **Table 1**, which illustrates how metric prioritization shifts across blood, biological fluids, and solid tissues.

**Table 1:** From guidelines to practice: Non-equivalence of relative and absolute metrics across biological compartments.

Compartment/Sample type	Metric with higher interpretative weight	Clinical implication/risk interpretation	Practical interpretative guideline
Peripheral blood	Relative (%) and Absolute (cells/µL) (dual reporting recommended)	Blood is a well-mixed compartment; percentages accurately describe composition when the total lymphocyte pool is stable, while absolute counts capture changes in total cellular mass during lymphopenia, immunosuppression, or immune reconstitution.	Always report both metrics. Prioritize absolute counts when total lymphocytes are reduced or clinically unstable. Avoid interpreting preserved percentages as preserved immune capacity.
Biological fluids (Cerebro-spinal fluid, ascites, serous effusions)	Percentage per volume (support with absolute counts)	Although fluids can be homogenized, the generally low cellularity often varies depending on the fluid type, ranging from paucicellular in basal conditions to pleocytosis in inflammatory effusions. Percentages may appear stable despite a minimal number of cells, leading to an overestimation of their biological significance.	Do not interpret percentages without volume-normalized counts or minimum event thresholds. Report processed volume and cellularity to contextualize proportional data. Absolute values ​​for subpopulations are generally not defined.
Solid tissues	Neither global % nor local absolute alone (phenotype/spatial context carries weight)	Spatial heterogeneity dominates; numbers reflect sampling site, not whole-organ immune status (e.g., lymphoid follicles, immune niches or tertiary lymphoid organs).	Interpret values as site-specific descriptors. Count the lymphocytes per 1 gram of tissue (cell density). Avoid extrapolating local percentages or counts to whole-tissue immune status without spatial context. The subphenotype of a population becomes more relevant. (e.g., B cell subsets or T cell subsets) Avoid overestimation caused by contamination of the tissue sample with blood-derived lymphocytes. (Use homing markers to identify tissue lymphocytes)
Any type of sample used in high-dimensional cytometry as an analytical context (e.g., mass or spectral cytometry)	Primary data architecture; relative (%) is the robust metric.	These studies prioritize phenotypic resolution and compositional analysis over absolute native quantification. The absence of intrinsic volumetric counting and the processing based on complex, non-canonical phenotypes make relative percentages within the acquired sample the fundamental and most robust metric.	Interpret primarily as relative architecture, heterogeneity, and inter-subpopulation relationships within the analyzed cell set. Do not equate high-dimensional % with clinical absolute counts. If you reconstruct absolutes (e.g., beads), state the method + limitations and frame as discovery-supportive, not definitive clinical quantification. Note that normal reference ranges have not been established for lymphocyte immunophenotypes with more than eight markers.

For the correct interpretation of the values ​​obtained, both as percentages and absolute values, it is necessary to guarantee analytical sensitivity, which is defined by three critical thresholds: the Limit of Blank (LOB), which represents the maximum expected signal in negative samples (statistically calculated with 95% confidence); the Limit of Detection (LOD), which identifies the minimum detectable concentration above the blank with a 5% error rate; and the Lower Limit of Quantification (LLOQ). The latter establishes the minimum level at which the measurement is not only detectable but also meets the standards of precision and accuracy necessary for clinical use, always being equal to or greater than the LOD. In this regard, for canonical TBNK immunophenotypes, existing recommendations specify a minimum of 2500 lymphocyte gate events and, for absolute counts on a single platform, no less than 1000 fluorescent counting beads. Furthermore, if the calibration factor is greater than 1000, then that will be the minimum possible value for accurate counting. It is also recommended to include quality controls that verify a lymphosum (sum of T, B, and NK cells) with a result between 95% and 105%, requiring review if it falls outside this range. Additionally, the sum of the percentages of CD4+ T cells, CD8+ T cells, double-negative T cells, and double-positive T cells should match the total CD3+ T cells ( ± 5%), while values ​​below the LLOQ should be reported as “less than” and trigger alerts for pathologist review.

### 3.2 Guideline on contextual relevance of immunophenotype

The interpretive references for an immunophenotype are not fixed but rather depend on the overall immune status in which it is evaluated and on interindividual variability. The exact value may be physiological, adaptive, or clinically relevant, depending on the biological context, inflammatory burden, systemic disease, or therapeutic response. In functionally intact immune systems, proportional variations usually reflect a precise modulation of the immune balance. Conversely, in pathological situations such as immunodeficiencies, advanced neoplasms, cytotoxic treatments, or transplants, even slight variations can acquire disproportionate importance.

Furthermore, interindividual variability influences the interpretation of immunophenotyping results. Factors involved in this interpretation include age, sex, genetic background, ethnicity, circadian rhythm, lifestyle, nutrition, toxic habits and medication use. This guideline formalizes a consensus, though not uniform, practice: interpretation is not based on an isolated number, but rather on the context in which that number arises and the reference range, if available. This assumption is rarely made explicit, and interpretation is often left to expert judgment.

Many of these sources of variability can be controlled in research, but not in clinical practice. This leads to a fundamental rule for all interpretations in the clinical setting: the interpretation of a lymphocyte immunophenotype must be accompanied by the patient’s clinical and sociodemographic data, the reference range alone is not conclusive.

These guidelines emphasize that immunophenotypic values ​​have no intrinsic biological or clinical significance in and of themselves. Their significance only arises when interpreted within the overall immune status and the specific biological or therapeutic contexts in which they are generated. A dichotomous “normal/abnormal” reading based solely on population ranges should be avoided. Instead, cytometric interpretation should adopt a logic of contextual normality, where the “expected” value is not a single value, but a probability space defined by biological and clinical determinants. Thus, “low” or “high” quantitative findings may be normal for a given individual if they are framed within interindividual variability and are not accompanied by converging signs of pathology. (**Table 2**).

**Table 2:** From guideline to practice: Context-dependent interpretation of lymphocyte immunophenotypes (clinical risk framing; technical causes excluded).

Clinical/Biological context	Key property	Clinical implication/risk interpretation	Practical interpretative guideline
Healthy individuals (reference setting)	Mild deviation from reference range	Usually low clinical risk; most often individual physiological variability shaped by age/sex/genetics, circadian rhythm, lifestyle, exposures, nutrition, and social determinants	Watchful waiting: do not label “abnormal” if isolated and asymptomatic; document context and re-check longitudinally (same conditions). Escalate only if clinical red flags or multi-parameter immune alteration appears.
Cancer & hematological malignancies (non-malignant lymphocytes)	Relative enrichment or depletion of normal T, B, or NK subsets	It often reflects context-driven immune remodeling (tumor burden, inflammation, stress, type of treatment or phase) rather than a primary immune pathology. (Non-pathological population)	Risk-based interpretation: interpret as a marker of immune status; act only if it aligns with a history of infections, systemic inflammation, frailty, or anticipated treatment toxicity. Otherwise, consider the trend rather than diagnosing. In selective lymphodepletion regimens, emphasize expected values. Consider the context-dependent dual role of some lymphocyte subsets such as Treg.
Inflammatory or infectious states	Increased proportion of activated or effector subsets	This response is usually appropriate in acute illnesses (defense, repair); in chronic settings, it may indicate ongoing immune activation, incomplete resolution, or exhaustion.	Phase pattern: In acute illnesses, treat as expected unless disproportionate; in chronic illnesses, require persistence + clinical congruence (symptoms/biomarkers) before labeling dysregulation; repeat after the resolution window. Avoid interpreting activation-associated shifts as pathological without systemic context.
Therapeutic procedures (chemotherapy, immunotherapy, transplantation, apheresis)	Preserved partially percentages with reduced absolute counts	There may be an increased risk of susceptibility despite a normal-appearing composition; changes can be expected depending on the regimen and its selectivity.	Immune reserve pattern: frame as reduced immune reserve when clinically coherent with timing/regimen; guide prophylaxis/monitoring by absolute counts and clinical course, not by percentages alone; avoid overcalling expected nadirs.
Post-therapy or immune reconstitution	Values approaching reference ranges	Near-normal may still be functionally insufficient; recovery is often asynchronous and exposure-dependent	Recovery pattern: declare recovery only with sustained stability in serial tests plus clinical adequacy (low infection burden, improvement of inflammation, post-transplant lymphocyte graft, appropriate response to vaccine if assessed); normalization at a single time point is not sufficient.

Therefore, the key practical guideline is in the absence of pre-analytical and analytical errors, interpret any deviation from the result as pathological only if it is incongruent with the individual context (age/sex/ethnicity, habits, comorbidities and drugs) and is accompanied by a convergent phenotype (symptoms, infections, inflammation or cytopenias); if the finding is isolated and the patient is clinically stable, treat it as individual normality and confirm with longitudinal follow-up.

### 3.3 Guideline on anatomo-phenotypic determination

An inherent limitation of FC, compared to tissue-based cytochemical methodologies, is the loss of topographic context. Except for peripheral blood or CSF, where cells are already in suspension in their native state, the dispersion required to obtain a cell suspension suppresses histological architecture and interactions (cell-cell and cell-stroma) in solid tissues.

The importance of immunophenotyping is intrinsically linked to the anatomical and functional compartment from which the sample is obtained. Direct extrapolation of values ​​between peripheral blood, cerebrospinal fluid, serous fluids, tissues, or cell products is not conceptually valid without prior interpretive reformulation, despite the well-known lymphocyte recirculation. Small quantitative or qualitative variations can reflect high-impact biological processes in compartments with limited cellularity or highly selective lymphocyte trafficking, such as immunologically privileged tissues. Furthermore, the absence or low representation of specific subpopulations is physiological and does not indicate dysfunction, such as the absence of B lymphocytes in cerebrospinal fluid. This guideline emphasizes that the compartment redefines the meaning of reference ranges, which must be understood as specific to the biological environment being analyzed. Moreover, lymphoid tissues contain several orders of magnitude more lymphocytes than other non-lymphoid tissues.

It is important to emphasize that the mere presence of a low-frequency population in each compartment should not, by itself, be considered pathological; its interpretation must be grounded in the biological context of that compartment and in analytically reliable quantification. In practice, this requires reporting the minimum event threshold and, where applicable, the assay-specific lower limit of quantification (LLOQ). In high-dimensional platforms or workflows with primarily architectural outputs, where absolute quantification is not intrinsic, these thresholds should be locally defined and validated according to the analytical design and intended use of the assay.

The lymphocyte immunophenotype is anatomical context-dependent. Identical phenotypic patterns may have distinct meanings depending on whether they arise in circulating blood, confined fluids, or spatially structured tissues (**Table 3**).

**Table 3:** From guideline to practice: Anatomical–phenotypic determination across compartments.

Compartment/Sample type	Key property	Clinical implication/risk interpretation	Practical interpretative guideline
Peripheral blood	Systemic level, well-mixed compartment with continuous lymphocyte recirculation	The best indicator of the circulating immune system, blood-derived reference ranges are valid internally only for blood. However, they are subject to variation due to regional and local changes in lymphocyte recirculation.	Use blood reference ranges only for whole blood. Indicate the lymphocyte immunophenotype of peripheral blood to complete the list for other compartments.
Bone marrow	Structured immune niche (not a homogeneous compartment): houses long-lived plasma cells and memory T lymphocytes and contains bone marrow-resident immune subgroups that are not reflected in the blood.	The proportion of mature lymphocytes in bone marrow is lineage-specific, as the count includes immature forms that express the canonical marker used for phenotyping. Samples (biopsy or aspirate) exhibit varying degrees of peripheral blood contamination. This overestimates the quantification of mature phenotypes. Differences between individuals are more pronounced in absolute values, while proportions are more stable.	Stratification by age and gender is essential in this type of sample. Absolute values ​​are essential because they detect changes more sensitively than percentages. Perform the count of the first pull obtained at a target volume of < 2mL to avoid overestimation due to hemodilution.
Cerebrospinal fluid (CSF)	Highly restricted compartment with immune surveillance–driven trafficking	Small numerical differences may reflect biologically relevant immune activation or pathology	Interpret low frequency but high biological weight populations with caution. Interpret as a paucicellular compartment without B lymphocyte representation.
Biological fluids (ascites, pleural effusions)	Semi-confined compartments influenced by local inflammation and tissue leakage	Immunophenotypes reflect both systemic immunity and local microenvironment	Interpret values as compartment-specific profiles. Avoid direct comparison with blood without explicit normalization strategy.
Solid tissues	Spatially heterogeneous immune microenvironments with focal lymphoid aggregates	Percentages and local counts reflect sampling site rather than organ-wide immune status. Tendency towards keeping excess contaminants: cellular debris, aggregates, fragments, and occasionally red blood cells.	Treat immunophenotypes as site-specific descriptors. Interpret the results based on the total cell yield. Use a selection strategy that excludes contaminants, combining viability staining with nuclear staining. Estimate the lymphocyte density per 1 gram of tissue and report both metrics % and cells/µL. Do not infer global tissue immunity without spatial or multi-site sampling context.
Apheresis or cell therapy products	Artificially generated and concentrated cell compartments, intended to achieve a harvested yield of specific cells.	The cell concentrations obtained reflect processing parameters and the biology of the target cell. The correct interpretation is to verify that the product yield allows reaching the target dose (Total cells or cells/kg) to be infused. Cryopreservation and thawing processes can introduce biases in the most sensitive cell populations in heterogeneous products.	Interpret phenotypes within the procedural context; avoid comparison with physiological compartments. Use an absolute cell count to calculate the dose to be infused and the percentage to estimate the weight or purity of the target population. Evaluate the viability of cell products by phenotyping the populations of interest on the overall assessment

### 3.4 Guideline on technical delimitation of the assay

The analytical architecture of a cytometric assay directly determines the types of inferences that can be legitimately made. The selection of markers, which ultimately defines the immunophenotype, the panel design, the gating strategy, the instrumental platform, and the quantification method all determine the dimensions of the immune system accessible for interpretation. Not all configurations allow for equivalent inferences; systems that enable intrinsic absolute counts offer a different perspective than those based solely on relative metrics, and high-dimensional platforms prioritize phenotypic resolution over direct quantification. This guideline reminds us that interpretation should not exceed the limits of the assay design and that conclusions should be formulated in accordance with the technical capabilities and limitations of the system used.

In addition to binary positivity-based gating, the density of marker expression may also carry relevant biological information. Accordingly, fluorescence intensity–based variables should be interpreted as a complementary semiquantitative layer of the immunophenotype, particularly in systems-level or high-dimensional analyses. However, such readouts should only support biological or clinical inference when their measurement is analytically standardized and stable, since fluorescence intensity is highly dependent on instrument configuration, compensation, reagent titration, and cross-run comparability. Thus, expression density may refine interpretation, but it should not be equated with functional status unless specifically validated for that intended use.

Furthermore, immunophenotyping assays can be classified according to their scope, origin, and regulatory control, as research use only (RUO) tests, *in vitro* diagnostic (IVD) tests, and laboratory-developed tests (LDT).

In a Research Use Only (RUO) test, the reagents and/or panel are intended for research and are not authorized for diagnostic use or clinical decision-making. Therefore, although they can be used to characterize immunophenotypic patterns and generate hypotheses, their results should not be presented as equivalent to a clinically validated assay. Guideline thumb: Interpret and report RUO findings as exploratory (descriptive or research) evidence, avoiding diagnostic thresholds or therapeutic conclusions unless formal validation/verification for the intended clinical use exists.

In an IVD test, the manufacturer designs, optimizes, and validates the assay, submits it for evaluation by a regulatory agency (e.g., the FDA), and, upon authorization, can market it as a kit for use across different laboratories. In that case, the user laboratory must follow the manufacturer’s Standard Operating Procedure (SOP) and verify locally that it reproduces the declared performance. A guideline of thumb is to interpret and report IVD results within the manufacturer’s intended use and avoid extending conclusions to matrices, populations, or endpoints not covered by their validation.

In contrast, a Laboratory Developed Test (LDT) is created, optimized, and validated by a specific laboratory using its own equipment, reagents, and personnel. In the US, these tests have traditionally operated under the CLIA framework, with some regulatory discretion, allowing laboratories to define and validate their own performance specifications. Once validated, an LDT can be applied to patient samples in that laboratory but is not sold as a kit to other institutions. An analogous scheme exists in the EU under the IVDR, which is also evolving. Guideline thumb: In laboratory testing (LDT), interpretation should be anchored to the performance specifications and ranges/thresholds defined by your own laboratory. If the context of use changes (sample, population, platform), the result should be treated as non-extrapolable until verification/revalidation.

Furthermore, the type of result reported determines the validation plan and subsequent statistical analysis. Generally speaking, assays can be quantitative, semi-quantitative, or qualitative. Quantitative assays rely on calibration and reference materials to provide numerical values (e.g., CD4 or CD34 enumeration by FC). Semi-quantitative assays produce estimates or magnitudes that do not depend on a classic calibration curve (e.g., MRI/MRD or PNH/PNH by FC). Qualitative assays report presence/absence or a descriptive interpretation rather than a numerical value, as is the case with diagnostic immunophenotyping for leukemia/lymphoma. Some trials combine several types of data (for example, identifying an abnormal population and also quantifying its percentage). Guideline thumb: interpret each output according to its nature in quantitative data, decide by absolutes/LOQ; in semi-quantitative data, by categories/trend; and in qualitative data, by positivity criteria, and do not convert a result to another type (e.g., inferring clinical significance from a percentage without quantitative support) if the trial design does not validate it.

An immunophenotype can be interpreted only within the inferential limits imposed by its analytical architecture; conclusions that exceed these limits constitute methodological extrapolation rather than biological knowledge (**Table 4**).

**Table 4:** From guideline to practice: technical delimitation of immunophenotypic inference.

Analytical architecture	What it truly measures (operationally)	Legitimate inference (what you *can* conclude)	Practical interpretative guideline
Marker selection (immunophenotype definition)	Identity and biological status. A construct defined by the exact marker set and positivity and negativity rules.	Only identity and states directly supported by the included markers are claimed; anything beyond that is speculative.	Base the interpretation primarily on consensus and canonical markers. Use the marker hierarchy: 1. Discriminator: live/death, nucleated cells, dump channel 2. Identity: backbone, essential, PAN, core lineage, sublineage, and maturation 3. Functional/State: activation, proliferation, exhaustion, trafficking, homing, polarization 4. Drop-in: exploratory, optional If any of 1–2 is missing, inference quality drops sharply (risk of ambiguity/artifact); 3–4 refine interpretation, but cannot rescue a poorly defined population. Do not base the interpretation solely on the percentage of positivity and expression level of the marker; also consider the expression pattern. Avoid inferring subsets by exclusion. If you do, do so only when using the marker that identifies the minority population, ideally when it represents <5%.
Panel design (breadth vs depth)	Breadth panels optimize coverage (many lineages, coarse resolution) Depth panels optimize resolution (fewer lineages, detailer state mapping). This choice sets which immune “dimensions” are observable (composition vs state architecture).	Breadth panels support robust inference about global composition/enumeration; depth panels support inference about phenotypic architecture (memory/activation trajectories) within the targeted compartment but not whole-system claims.	State in advance the primary endpoint(s) your panel is designed to address (enumeration vs. mapping of states) and interpret only with that resolution. Do not infer mechanisms from screening panels or claim system-wide immune status from a comprehensive panel focused on a single lineage. (Clinical validation guidelines emphasize the suitability of the panel/gate for their intended use.)
Gating strategy	The analysis algorithm converts raw events into reportable numbers (%, cells/µL, MFI). Gate variability can be an independent source of variation even with identical data.	You can infer differences only to the extent that the gating strategy is stable, auditable, and appropriate for the population definition; biological differences cannot be asserted when results are gate-sensitive. In secondary or tertiary markers, the cutoff point set in the selection gate is critical in quantitative measures and therefore a source of inaccuracies.	Prioritize the use of validated and consensus-based gate strategies if available. Do not modify them if they will produce the same results for which they were designed. Do not adjust cutoff levels once they have been set using the correct procedures. When heterogeneous results are expected, do not use the software’s automatic gating features.
Quantification method (Absolute counts vs relative-only)	Absolute counting (volumetric or bead-based, single-platform) measures cell concentration per unit volume; relative-only measures composition within the acquired set.	Absolute methods allow inference about immune reserve/risk and reconstitution; relative-only supports inference about relative shifts but cannot establish preserved/declined immune mass.	This was already addressed in guideline 1. Only add that interpretations of absolute counts are more recommended on a single platform than on a dual-platform.
Instrument platform (conventional vs spectral vs mass)	Each platform has its own specific measurement space, configured by optics/detectors, compensation vs. demixture, sensitivity, and dispersion error; outputs between instruments are not inherently interchangeable without harmonization. Mass cytometry lacks native absolute quantification; reliance on relative metrics	Only may compare results within a harmonized instrument/QC framework; cross-platform or cross-site equivalence requires explicit standardization/bridging, otherwise observed differences should be presumed method- or platform-driven rather than biological.	Interpret based on detailed knowledge of the platform used. (e.g., mass cytometry does not provide absolute counts; cytometers with specific fluidics systems are capable of delivering absolute counts without the use of counting beads; spectral analysis natively eliminates errors due to spectral overlap). Document the platform, configuration, and quality control approach, and restrict interpretations to the validated domain (site/instrument/panel). Mass cytometry is not yet validated for use in clinical practice.
High-dimensional cytometry	Maximizes phenotypic resolution and relationship mapping (clusters, trajectories) within the analyzed cell set; absolute quantification is not intrinsic unless specifically engineered (e.g., counting strategy) and validated for that use-case.	The interpretation is fundamentally architectural: heterogeneity, regularities, associations, and phenotypic structures. For research, this is excellent, but for translation to clinical cut-off points, top-down mapping/validation in clinically quantitative frameworks is required.	The interpretation is based on a structured analysis. Biology is interpreted only after ensuring a reproducible pipeline (14): pre-process and clean (singlettes/viable samples, artifact exclusion) (25), transform and normalize data (e.g., arcsinh; and batch correction where applicable), (3) use dimensionality reduction (t-SNE/UMAP/PacMap, etc.) primarily for visualization/global structure (not for quantification) (7), define populations with clustering in marker space (FlowSOM/PhenoGraph or others; clustering does not necessarily depend on dimensionality reduction), and (5) validate stability (parameters, randomization, iterations, consistency between runs/batches) before drawing conclusions; if the finding changes when reasonably varying parameters/iterations or repeating integration/batch correction, treat it as an artifact of the analysis and not as a biological signal.

### 3.5 Guideline on analytical and interpretative stability

Once the inferential boundaries imposed by the analytical architecture are recognized, the remaining challenge is to ensure that the observed variation reflects biological rather than analytical instability. Analytical variability is not only a source of random noise but also a factor that can introduce interpretive drift when it accumulates systematically throughout the preanalytical and analytical processes.

Before attributing clinical or biological significance to marginal differences, it is essential to distinguish biological immune variation from analytical variability, because the observed variation in laboratory or analytical results can stem from both sources. Biological variation refers to the natural fluctuation of a measurand (e.g., a biomarker, immunotype, or analyte) around an individual’s homeostatic set point. Analytical variability, on the other hand, includes variations introduced by the measurement process, instruments, reagents, and procedures, and is not related to the biological state of the subject.

To ensure that variations reflect biological findings rather than instability, it is imperative to understand and quantify these sources separately. Observed variability should be attributed to biological processes only after analytical stability has been established; otherwise, the variation must be interpreted as noise rather than a signal. (**Table 5**).

**Table 5:** From guideline to practice: analytical stability and noise control in immunophenotyping.

Source of variability	Mechanism of noise introduction	Impact on interpretation	Practical control guidelines
Pre-analytical handling	Pipetting impressions. Cell loss, selective depletion, and impairment of viability during washing, centrifugation, and filtration; changes dependent on the number of steps, time, and temperature, especially during prolonged processing.	Falsely low absolute counts and disproportionate loss of fragile/rare subsets apparent depletion or disappearance of low-frequency populations	Minimize handling steps; standardize processing time and temperature. For reliable counts, use reverse pipetting or positive displacement pipettes. Use lyse-no-wash or no-lyse-no-wash protocols as possible. Protocols that include centrifugation/washing do not necessarily perform an actual cell count of the sample; they tend to perform recovered cell counts (recovered cells/µL), unless consistent recovery is demonstrated.
Instrumental variability	Laser drift, detector sensitivity changes, suboptimal compensation, instability of the acquisition	Apparent changes in expression pattern, phenotypic and/or concentration unrelated to biology	Enforce daily QC and apply harmonized instrument settings. Include global fluorescence controls and a dot plot that monitors the stability of the acquisition over time.
Event acquisition thresholds	Too few total events in low-cellularity/rare-population settings	High stochastic error, unstable percentages, and unreliable detection of rare subsets	Do not over-interpret low-frequency findings unless the minimum number of events is met; define and report the minimum number of events and a population-specific lower limit of quantification (LLOQ) and classify results below threshold as detected but not quantifiable (or not reliable for quantification).
Absolute counting architecture	Bead recovery inefficiency or volume inaccuracies	Systematic bias in absolute values	Validate bead performance and volume accuracy; reject runs failing QC criteria, instability in acquisition of beads, or a low number of beads are acquired. (less than 1000)
Workflow complexity	Multi-step protocols with operator-dependent variability	Accumulation of small errors into large interpretative deviations	If the research or clinical endpoint depends on quantification, default to the simplest standardized workflow (automation where possible) and pre-validated reagents (e.g., dry polychromatic tubes); treat results from complex, non-standard workflows as lower-confidence unless run-to-run controls confirm stability.
Staff subjectivity	Operator staff: non-standardized training, limited supervision, and inconsistent instrument handling (setup/QC, thresholds, gate placement), compounded by variable software proficiency and undocumented workflow changes. Analyst staff: non-standardized training and inconsistent interpretive decisions (gating, classification, international consensus), particularly among personnel responsible for clinical interpretation and result report.	Introduce systematic and stochastic errors, undermining longitudinal/multicenter comparability and inflating misclassification risk, particularly near technical limits and in rare-event populations.	Establish an auditable competency framework: role-specific qualification criteria; documented initial and periodic competency assessments (including proficiency testing for gating/interpretation for reporting staff); formal authorization for critical activities (method changes and result release); and continuous education. Standardize the SOPs. External senior staff certification may be used as an additional harmonization layer. Ensure a minimum number of exposures to new clinical cases per year. R packages such as CytoGMM can be used, which accurately reduce biases arising from correlations between markers and protect against false discoveries induced by patient heterogeneity.

Finally taken together, these guidelines provide a structured framework for the critical evaluation of normal lymphocyte immunophenotypes in complex contexts. Far from replacing existing technical guides or diagnostic frameworks in cytometry, these guidelines explicitly state interpretive principles that govern expert practice, facilitating their transfer, discussion, and critical evaluation.

## 4 Discussion and perspectives

FC has achieved a high degree of technical maturity, with detailed guidelines for experimental design, data acquisition, and analysis, as well as analytical quality criteria. However, this methodological standardization has not been accompanied by an equivalent formalization of the interpretative processes that mediate between cytometric data and immunological inference. As a result, technically correct results can lead to divergent interpretations when applied in different biological contexts, when unconventional compartments are analyzed, or when heterogeneous analytical architectures are used to study samples.

This study addresses a gap in cytometry immunology: the disconnect between FC’s high technical reproducibility and limited interpretative reproducibility of lymphocyte immunophenotypes. The gap stems not from insufficient data or technology, but from lacking explicit interpretive principles. By formalizing guidelines for immunophenotypic interpretation non-metric equivalence, context-dependent interpretation, anatomical-phenotypic determination, technical delimitation, and analytical stability - this framework shows how heterogeneity across studies can be understood as a structured property of immune system measurement rather than uncontrolled variability.

In immunophenotypic studies, a rule of thumb for immunologists is to use absolute counts when the precise quantification of specific cell subsets is critical. Because they are not affected by changes in other co-evaluated cell populations, in proportional terms, and provide a more reliable assessment of specific target cells. For example, in the diagnosis of hematological diseases, prognosis of diseases affecting immune system homeostasis, and in the monitoring of cell therapies. Specifically, we can cite the CD4+ T cell count in HIV patients or other immunodeficiencies, the evaluation of lymphocyte subsets for cancer, or lymphocyte engraftment during follow-up after hematopoietic stem cell transplantation (26–31). Its main limitation is that normal ranges are not well established in most tissues, except for peripheral blood.

Percentage counts provide more precise and reproducible data due to narrower expected value ranges compared to absolute counts of lymphocytes and leukocytes. Its main limitation is that normal percentages can mask abnormal absolute counts. Reporting both metrics (% and cells/µL) is most informative and will help establish reference values. Percentages are better for evaluating relative distribution of immune cell subsets, useful for understanding immune response dynamics (21, 22, 30). For instance, the percentage of markers in cerebrospinal fluid can indicate prognosis, independent of absolute counts (32).

The scientific evidence consistently shows that relative and absolute metrics capture different biological dimensions, that the meaning of a value depends on the overall immunological state, and that the anatomical compartment redefines the relevance of the observations (8, 21, 33). Nevertheless, these notions are rarely articulated together, fostering implicit extrapolations and conceptually fragile comparisons between studies.

The percentages (%) reflect the relative proportion of a given immune cell subset within a reference population, such as total lymphocytes or white blood cells. For example, a FC immunophenotyping assay may report the percentage of CD4^+^ T cells among total lymphocytes or among T lymphocytes. This relative measure is useful for understanding the composition of the immune profile and highlighting shifts in cell subset proportions. Absolute counts (cells/μl) provide the exact number of cells per unit volume (21).

Clinical studies emphasize that absolute counts often provide more reliable prognostic and diagnostic information than percentages alone. Absolute counts are crucial for assessing immune competence because a low absolute count, even if the percentage is normal or elevated, may indicate immunodeficiency or pathology. For example, in HIV infection, absolute CD4^+^ T cell counts (cells/μL) are the standard marker of immune status, monitoring and guide therapy, whereas CD4^+^ percentage alone can be misleading, especially in conditions that affect total lymphocyte counts (34–37). The novelty of this guide lies in formalizing the prioritization of the metric when interpreting results, considering the sample context and condition or disease, and treating the %/absolute disagreement as a biological signal rather than an artifact. Of course, this applies only if it occurs and the method used is validated and has the appropriate quality control approved.

In sepsis, the absolute count of lymphocyte subpopulations allows patients with sepsis to be classified according to their risk of mortality and contributes to clinical decision-making (38). In SARS-CoV-2 infection, CD4^+^ lymphocyte absolute counts identify patients at increased risk of unfavorable outcomes (39). Similarly, in natural killer/T-cell lymphoma, low absolute CD4^+^ counts are associated with worse overall survival, signifying prognostic value beyond percentage-based data (40). It has also been shown that, when assessing immune reconstitution after allogeneic hematopoietic stem cell transplantation, absolute counts of T lymphocyte subsets are clinically relevant (41), and in the case of autologous autograft infusion, absolute numbers of immune effector cells directly impact clinical outcomes (42).

The EuroFlow consortium developed the PID orientation and screening tube for standardized immunophenotypic diagnosis of lymphoid PIDs, enabling data exchange between centers. While reporting results in both metrics and automating analysis, it lacks clear quantitative interpretation guidelines, focusing on multidimensional distribution (43, 44). High-dimensional analysis using 40–50 markers, automation, and AI has led to methodologies for automated data acquisition, analysis, and reporting. Though valuable, these methods do not establish guidelines for biological and clinical interpretation of AI-generated results (45).

There can be discordance between the percentages and absolute counts in diseases that alter total lymphocyte or white blood cell numbers. For example, patients with cirrhosis show normal CD4 percentages despite low absolute CD4+ counts, likely due to splenic sequestration and portal hypertension, which cause low circulating lymphocytes despite stable relative proportions (46). This illustrates that relying solely on percentages can mask clinically significant absolute cell deficits.

​In oncology, absolute lymphocyte subset counts (CD3+, CD4+, CD8+, B, and NK cells) serve as biomarkers to predict cancer progression and prognosis. Lower absolute counts indicate immune impairment and are associated with shorter progression-free survival, making them useful for clinical decision-making and evaluating treatment efficacy (27, 29, 47, 48). In chronic conditions such as end-stage renal disease with hemodialysis, absolute counts reveal profound lymphocytopenia and quantitative immune alterations that percentages alone cannot detect, helping to explain disease-associated immune dysregulation (49).

Overall on this guideline, absolute immune cells count is preferred when the total number of immune cells affects clinical risk stratification, prognosis, and therapeutic decisions. Percentages may mask clinically important lymphopenia or immune depletion if total cell counts fluctuate, making absolute counts more reliable in immunodeficiency, cancer prognosis, sepsis, immunotherapy monitoring, and transplant medicine. These measures are complementary and should be interpreted together for a comprehensive assessment of immune status.

The interpretation of lymphocyte immunophenotypes requires consideration beyond numerical values, as they are influenced by immunological context. Factors including biological background, inflammation, diseases, therapies, lifestyle, living area, nutrition, age, and sex shape lymphocyte profiles (50–54). Age determines immunosenescence and chronic inflammation, while sex differences affect lymphocyte populations: women show stronger pro-inflammatory responses with increased lymphocyte activity, whereas men have expanded regulatory subsets (51, 52). Aging influences lymphocyte function differently in men and women, affecting disease susceptibility and requiring sex-specific interpretations (50–54). This approach prevents misinterpretation and enables tailored immune assessments.

Within the Guideline on Contextual Relevance of Immunophenotype, a critical point is recognizing that some cell populations, such as Tregs, have a dual functional role whose biological and clinical significance depends on the disease. Even within the same pathology, such as cancer, the role will depend on the tumor type, the compartment analyzed (tumor vs. stroma vs. blood), and the inflammatory landscape. In tumors where a strongly immunosuppressive microenvironment predominates, an increase in Tregs often constitutes an evasion mechanism and has been associated with a worse prognosis; this has been described in ovarian, breast, melanoma, gastric, and non-small cell lung cancers. In contrast, in colon and head and neck carcinomas, a paradoxically favorable association with Tregs has been reported (especially in certain contexts, such as HPV^+^ status and/or stromal location), suggesting that, in some scenarios, Tregs may act as a marker of an organized immune microenvironment or effective inflammation rather than simply suppression (55–58). Therefore, the same direction of phenotypic change (**↑**Treg) does not constitute a conclusion in itself: it must be interpreted in a contextualized way (tumor entity, tumor/stromal anatomy, quantification method, and clinical covariates), and the discussion must make explicit that interpretative rule to avoid extrapolations between diseases in which the biomarker changes meaning.

The novelty of this guideline lies not only in recognizing that age, sex, or disease modify the interpretation of the immunophenotype, but also in transforming this variability into an explicit logic for the clinical interpretation of risk and management. In this sense, the value of lymphocyte populations ceases to be an isolated or dichotomous figure (within or outside the reference ranges) and is understood as a finding whose meaning depends on the overall immune status, the therapeutic context, and the patient’s clinical congruence. Its distinctive contribution is that it operationalizes this “contextual normality” into rules for interpretive decision-making: it avoids the overdiagnosis of mild findings in healthy individuals, redefines deviations in cancer as signs of immune remodeling rather than primary pathology, allows for the recognition of patterns of depleted immune reserve after lymphodepleting therapies, and requires serial stability and clinical adequacy before declaring immunological recovery. In this way, the guideline transforms the context into a structured criterion for clinical decision-making, rather than a mere general interpretive warning. This presupposes that clinical data, in the form of an immunophenotyping request for lymphocyte population counts, are required.

The anatomical immunophenotypic determination for lymphocyte immunophenotyping by FC depends on the sample source, whether blood, cerebrospinal fluid (CSF), ascites, or solid tissues (lymphoid or not lymphoid organs), owing to differences in cellular composition, viability, and immunophenotypic characteristics across these compartments.

In peripheral blood, flow cytometric immunophenotyping is well established, with detailed reference values available for various lymphocyte subsets, such as CD4/CD8 T cells, B cells, and NK cells. Blood samples typically contain abundant lymphocytes with high viability, facilitating robust, reproducible phenotyping, including the use of cryopreserved samples for epidemiological studies without significant loss of accuracy. This stable cellular environment supports routine diagnostic and monitoring applications in hematological and immunological disorders (6, 14, 15, 18, 21, 50).

In contrast, CSF samples pose unique challenges due to their paucicellularity and rapid decline in cell viability after collection (59). Despite this, FC-based immunophenotyping of CSF is crucial for detecting hematologic malignancies, such as lymphoma and leukemia, that infiltrate the central nervous system (CNS). Multiparameter FC significantly enhances sensitivity compared to traditional morphological and cytological examinations. Furthermore, CSF lymphocytes show different functional and phenotypic profiles compared to blood; for instance, CNS-infiltrating lymphocytes can exhibit distinct subsets, such as the predominance of CD8 T cells in the parenchyma versus CD4 T cells in CSF, reflecting compartment-specific immune responses (59–61).

Ascitic fluid or other bodily fluid samples also present variable lymphocyte populations with distinct immunophenotypic profiles, which require tailored antibody panels and gating strategies for the accurate detection and classification of lymphoid proliferations or inflammatory conditions. These samples typically have lower cellularity and greater variability than blood, requiring optimization of sample handling and analysis settings (62).

Solid tissue samples, including those from the spleen or lymph nodes, are highly complex and differ from peripheral blood samples. Immunophenotyping must account for diverse lymphoid architecture, cellular microenvironments, and mixtures of mature and immature lymphocytes, as well as stromal and accessory cells (33). The most important rule when interpreting data is to start with a selection window strategy that excludes major sources of error, such as contaminants: cell debris, aggregates, and fragments, which affect cell performance by causing underestimations (63). FC panels for solid tissues are used to study lymphoid tissues (composition, lineage, maturation), differentiate reactive from neoplastic lymphoid populations in lymphomas, and analyze the microenvironment in solid tumors. This latter application facilitates the classification of tumors according to the type and degree of immune infiltration into immune-desert, immune-exclude, and immune-inflamed, which is crucial for immunotherapy strategy (64).

With the development of cell transplantation and cell therapies, the use of cryopreserved products is increasing. In these cases, cell viability analysis is performed before use, both for research and clinical therapies. However, in heterogeneous cell products, such as leukapheresis, this analysis alone does not guarantee the preservation of the target subgroups for our purposes, since some populations may be more susceptible to cryogenic or thawing processes. For this reason, it is necessary to assess viability by adding markers for the immunophenotype of interest, rather than globally (65, 66).

In summary, the anatomical site of sample acquisition critically influences the immunophenotypic determination of lymphocytes using FC. Blood is a well-characterized and abundant cellular source with standardized reference values, whereas CSF and other body fluids require enhanced sensitivity strategies owing to low cell counts and viability issues. Solid tissues require more complex antibody panels and analyses to find heterogeneous cell populations within tissue architecture. These differences underscore the importance of sample-specific protocols and interpretation of lymphocyte immunophenotyping to ensure accurate diagnostic and research results.

The novelty of this guideline lies in its transformation of the anatomical compartment from a mere source of variation into a formal determinant of the clinical significance of the immunophenotype. It does not simply point out that blood, CSF, or tissue differ, but establishes that each compartment redefines the reference range and the very meaning of the finding. **Table 3** translates this into operational rules: blood as a reference valid only for blood, bone marrow with first-pull and hemodilution control, CSF as a paucicellular compartment of high biological weight, solid tissues as site-specific descriptors, and apheresis or cell therapy products as procedural compartments oriented toward dose and purity. Thus, the novelty lies in proposing a true anatomy of interpretation, and not just of the sample. These guidelines become even more relevant when reference ranges are unavailable for most of the samples that could be clinically studied.

Likewise, the proposed guidelines emphasize that the technical architecture of an assay is not interpretively neutral. The capacity for inference is limited by the panel design, instrumental platform, and quantification method employed. Recognizing these limitations does not weaken the analysis; rather, it strengthens the validity of the conclusions by aligning inferences with what the analytical system can support.

​Studies have emphasized that a single laboratory result represents a distribution influenced by both biological and analytical variations. In this sense, guideline 4 connects with guideline 5 in the second aspect of analytical variability with analytical and interpretation stability. The analytical component is primarily influenced by the operator’s expertise and certification and lies fundamentally in the self-instrument settings and the self-interpretation of the data. Understanding these components facilitates the interpretation of clinical or experimental data, especially when the analysis is performed manually, in quantitative and semi-quantitative measurements, or when the values ​​approach reference limits or clinical decision thresholds (67–69).

Disentangling biological variation from analytical variability requires careful experimental design and statistical analysis. For example, nested variance analysis models can separate intra-individual biological variations from analytical imprecision by performing replicate measurements and repeated sampling over time. Increasing the number of replicates can reduce the impact of analytical imprecision, and adequate sampling strategies can improve the reliability of estimating biological variations (70, 71).

Analytical precision should be optimized through rigorous quality control and standardized protocols. The observed variation must be compared with known analytical variability thresholds to determine whether the changes exceed what could be expected by the measurement error alone. For example, one of the most significant sources of analytical and interpretive variability lies in the design of the gating strategy, and one solution was the use of a predefined analysis protocol, which reduced variability by 92% (69).

A strategy to reduce analytical variability and contribute to the stability of interpretation in the quantification of lymphocyte subsets by FC is to simplify the technical procedure, reducing complexity, time, steps, and sources of error. This improves the Method Design Practicality Criteria of “Ease of Use,” “Length of Method,” and “hands-on time” (71). Another strategy is to automate data analysis and use artificial intelligence and machine learning algorithms (supervised and unsupervised methods), thereby considerably reducing variability in analysis and interpretation (45, 72, 73).

Guidelines 4 and 5 offer a convergent clinical innovation by establishing that the utility of immunophenotyping depends not only on generating technically correct data, but also on demonstrating that the inference is compatible with the assay architecture and resistant to analytical instability. What appears obvious in an individual case becomes novel when transformed into an explicit framework of generalizable clinical validity. Thus, the proposed framework makes technical delimitation and interpretative stability prerequisites for the clinical reading of the result: what the system is not designed to measure should not be inferred, and what does not remain stable in the face of analytical noise should not be translated into biological meaning or a clinical decision. In this way, both guidelines transform methodological control into an explicit safeguard against diagnostic extrapolation, overinterpretation of marginal differences, and the clinical use of findings that may still constitute artifacts rather than signals. Objectively, it is recommended that whoever interprets and reports the result consider that the finding remains within the assay’s validated scope and does not exceed the expected analytical variability of the method.

Some important elements to consider when applying guidelines 1, 3, 4, and 5 to the interpretation of results are the LLOQ and the minimum number of events required for the quality of the data obtained. For TBNK immunophenotyped cells, these were already addressed in guideline 1 according to Assay Development and Validation of T, B, and NK Lymphocyte Subset Enumeration (ICCS, 2024) (23). In EuroFlow methods, populations represented by <50 events are often considered outside the quantitative range of the assay. In high-sensitivity rare event applications, such as PNH assessment, the LLOQ for RBCs is 0.01%, for neutrophils between 0.01% and 0.02% depending on the number of neutrophils acquired, and for monocytes is variable (0.1%-1%) depending on the number of monocytes available. In minimal residual disease assessment, 20–30 events can support detection/presence (LLOD), while >50 events are commonly accepted as the minimum threshold for reproducible quantification, and a target of 100 events is associated with an approximate counting imprecision of 10% (74–76). These are clear examples of elements that can affect the technical delimitation of the inference.

High-dimensional flow cytometry and artificial intelligence do not diminish the need for an interpretive framework; on the contrary, they make its practical value even more evident. In this context, our proposal specifically improves AI-assisted analysis. First, the guideline on the technical delimitation of inference prevents clusters, embeddings, or classifications derived from the algorithm from being interpreted beyond the validated marker set, the assay design, the correct cytometer setup and quality control, and the intended clinical use. This is especially important given that high-dimensional workflows are highly dependent on preprocessing options, normalization, batch correction, and clustering strategy. Second, the guideline on the non-equivalence of metrics helps avoid a common translation error in AI-driven flow cytometry: treating compositional results or cluster frequencies as if they were equivalent to absolute immune reserve. Third, the guideline on anatomophenotypic determination prevents the assumption that the same computational phenotype has the same significance in blood, cerebrospinal fluid, tissues, or cell products. Technical delimitation of inference requires that computational outcomes remain within the validated scope of the panel, platform, and intended use, and that dimensionality reduction primarily serves visualization and clustering methods that define candidate populations in the marker space; Analytical and interpretative stability requires that such phenotypes remain robust during preprocessing, batch correction, parameter variation, and repeated runs before they are assigned biological or clinical significance.

AI algorithms must be trained and validated specifically for each compartment, since the composition and interpretive significance of lymphocyte subsets differ between blood, cerebrospinal fluid, tissues, and cell products. Furthermore, a clinically relevant implementation should incorporate an absolute count strategy or validated linkage to absolute count data to allow for the parallel interpretation of relative and absolute metrics, rather than relying solely on compositional frequencies. Therefore, rather than conflicting with modern systems immunology workflows, this framework provides the interpretative conditions that allow standardized and validated computational phenotypes to be translated into clinically consistent immunological inferences.

Finally, the guideline on analytical and interpretative stability provides a concrete validation rule for AI workflows: findings should only be considered clinically relevant if they remain reproducible under reasonable variations in preprocessing, parameter settings, and batch integration. Therefore, rather than simply coexisting with new technologies, this framework functions as the interpretive layer that makes AI-assisted high-dimensional cytometry more clinically reliable, transferable, and biologically defensible.

​In summary, separating biological variation from analytical instability requires the following:

1. Recognizing inherent biological fluctuations in individual homeostasis.
2. Characterizing and minimizing analytical variability via quality control and standardized procedures, with reduced and automated steps in the preanalytical and analytical phases.
3. Using proper statistical designs to independently estimate biological variation.
4. Applying thresholds (e.g., reference intervals) to interpret the observed changes beyond the expected measurement noise.
5. Implement staff supervision and monitoring, along with an auditable competency program that includes role-specific qualifications, regular competency testing, formal authorization for critical tasks, and ongoing training. For senior staff, require international certifications whenever possible.
6. To address the problem of variation and separate technical noise from the biological signal during results interpretation, the R package CytoGLMM can be used. This analytical framework employs generalized linear mixed models (GLMMs) that allow inter-subject heterogeneity and batch-to-batch variations to be modeled as random effects, thus isolating the effect of experimental or clinical conditions as fixed effects. Applying this methodology, especially to immunophenotyping panels, ensures that detected differences in marker expression or cell abundance reflect genuine biological changes and not artifacts resulting from the inherent technical variability of flow cytometry (77).

Failure to consider these aspects can lead to interpretive drift and misattribution of variation, thereby compromising reliable biological inferences. Hence, it is essential to address both the analytical precision and biological variability.

In general, the framework’s contribution is especially focused on interpreting results obtained solely through numerical values ​​using a properly defined and clinically validated method. What seems “obvious” to a highly experienced specialist in cytometry or immunologist ceases to be so when the case is in a gray area, when the specialist is an early-career, when the compartment changes, when the panel was not designed for a specific inference, when there is a low number of events, or when the result must be read by another laboratory, another clinician, or an automated pipeline. The framework’s value lies in transforming this expert intuition into a reproducible interpretive logic, precisely to prevent heterogeneity from resulting in inconsistent reports or decisions.

As a practical complement to the present framework, we also propose an interpretive reporting template, provided as supplementary material, to support context-aware and clinically meaningful reporting of lymphocyte immunophenotyping results beyond the isolated presentation of numerical values. (Suplmentary document 1).

A necessary next step will be to stress-test the present framework in public high-dimensional datasets and real-world clinical cohorts, in order to determine whether its explicit application modifies biological conclusions, improves interpretive reproducibility, and reduces false-positive inferences arising from metric misuse, compartmental extrapolation, assay overreach, or analytical instability (78).

## 5 Conclusions

The lymphocyte immunophenotype is not a static object or a universal descriptor of the immune system but rather a contextual representation whose significance emerges from the interaction between biological properties and analytical conditions. Throughout this study, we have shown that much of the heterogeneity observed in FC studies does not reflect a lack of technical rigor or intrinsic biological inconsistency, but rather the absence of explicit principles to guide the interpretation of immunophenotypic data. By formalizing five interpretative guidelines: metric non-equivalence, context-dependent interpretation, anatomo-phenotypic determination, technical delimitation of inference, and analytical stability- this framework offers a conceptual structure that enables the technical reproducibility achieved by modern cytometry to be aligned with the interpretative reproducibility in immunological inference.

Far from reducing the complexity of the immune system, this approach recognizes heterogeneity as an informative property, provided it is understood within a suitable framework of the immune system. Ultimately, the value of this framework does not lie in proposing new markers, technologies, or cellular classifications, but in making explicit how we think about immunophenotypes. By shifting the focus from the search for universal reference intervals to a situated interpretation, this study aims to contribute to a more coherent, comparable, and conceptually mature application of FC to the immune system, capable of integrating health, disease, and therapeutic procedures without losing rigor or meaning.

## Data Availability

The original contributions presented in the study are included in the article/**Supplementary Material**. Further inquiries can be directed to the corresponding author.
