# Supplementary material for: A context-aware interpretive framework for lymphocyte immunophenotyping by flow cytometry
Source: Front Immunol. 2026 May 29;17:1788361. doi: 10.3389/fimmu.2026.1788361 (PMC13260266; doi:10.3389/fimmu.2026.1788361)
Supplement: Supplementary file 1 [file Table1.docx]

**PROPOSED INTERPRETIVE REPORT TEMPLATE**

Flow Cytometry-based Lymphocyte Immunophenotyping

*Operational fill-in form aligned with the framework principles*

| **1. Administrative and patient data** | |
| --- | --- |
| Report ID: ______________________ | Date: ______________________ |
| Patient ID / initials: __________________ | Age / Sex: _______________________ |
| Requesting physician: __________________ | Laboratory / site: __________________ |
| Clinical indication / diagnosis  _______________________________________________________________________________________________ | |
| Relevant current context  ☐ Healthy / reference setting ☐ Infection / inflammation ☐ Cancer ☐ Immunodeficiency ☐ Post-therapy / reconstitution ☐ Transplant ☐ Cell product ☐ Use of specific immunomodulatory drugs: Date of last dose: ________________  ☐ Other: ________________________________________________________ | |
| Relevant therapy / exposure: _______________ | Prior comparable study available: ☐ No ☐ Yes Date __________ |
| **2. Specimen and compartment** | |
| Specimen / compartment  ☐ Peripheral blood ☐ Bone marrow ☐ CSF ☐ Serous / ascitic fluid ☐ Solid tissue ☐ Apheresis / cell therapy product ☐ Other | |
| Anatomical site / source: __________________ | Collection date & time: _______________ |
| Processing start time: ___________________ | Processed volume / yield: _________________ |
| % of cellular viability: ___________________ | Anticoagulant used:: _________________ |
| Compartment-specific note  _______________________________________________________________________________________________ | |
| **3. Assay architecture** | |
| Assay type: ☐ IVD ☐ LDT ☐ RUO | Platform: ☐ Conventional ☐ Spectral ☐ Mass |
| Quantification: ☐ Single-platform absolute ☐ Dual-platform absolute ☐ Relative only | Panel objective: ☐ Enumeration ☐ Subset distribution ☐ State mapping ☐ Product characterization |
| Panel / reagent code: __________________ | Reference interval source: ________________ |
| Rationale for indicator selection  _______________________________________________________________________________________________  _______________________________________________________________________________________________ | |

| **4. Analytical quality and variation control** | | | |
| --- | --- | --- | --- |
| **Analytical variation control** | **Status** | **Analytical variation control** | **Status** |
| Specimen suitability acceptable | ☐ Yes ☐ No ☐ N/A | Processing time within validated window | ☐ Yes ☐ No ☐ N/A |
| Daily instrument QC passed | ☐ Yes ☐ No ☐ N/A | Compensation / unmixing acceptable | ☐ Yes ☐ No ☐ N/A |
| number of events acquired | _____________ | Absolute count QC passed (if applicable) | ☐ Yes ☐ No ☐ N/A |
| Analyst review completed | ☐ Yes ☐ No ☐ N/A | Method comparable to prior study | ☐ Yes ☐ No ☐ N/A |
| Target population below the LLOQ, interpret with caution | ☐ Yes ☐ No ☐ N/A | Pass Lymphocyte Sum Check (T+B+NK)  *(Acceptable range: 95% - 105%)* | ☐ Yes ☐ No ☐ N/A |
| Relevant analytical limitations / pre-analytical concerns  _______________________________________________________________________________________________ | | | |
| **5. Results summary** | | | |

*Fill the “Priority metric” field as Rel, Abs, or Both according to the intended interpretation.*

| **Population / subset** | **Relative (%)** | **Absolute** | **Reference / target** | | **Flag** | **Priority metric** | **Brief note** |
| --- | --- | --- | --- | --- | --- | --- | --- |
| ______________ | ________ | ________ | ________ | | _____ | _____ | ________ |
| ______________ | ________ | ________ | ________ | | _____ | _____ | ________ |
| ______________ | ________ | ________ | ________ | | _____ | _____ | ________ |
| ______________ | ________ | ________ | ________ | | _____ | _____ | ________ |
| ______________ | ________ | ________ | ________ | | _____ | _____ | ________ |
| ______________ | ________ | ________ | ________ | | _____ | _____ | ________ |
| ______________ | ________ | ________ | ________ | | _____ | _____ | ________ |
| ______________ | ________ | ________ | ________ | | _____ | _____ | ________ |
| Comments: Add aspects related to the expression pattern of the markers or expression density, median fluorescence intensity, or other aspects to highlight in this area.  _______________________________________________________________________________________________  _______________________________________________________________________________________________  _______________________________________________________________________________________________  _______________________________________________________________________________________________ | | | | | | | |
| **6. Integrated interpretation** | | | | | | | |
| Dominant interpretive frame  ☐ Composition shift ☐ Reduced immune mass ☐ Expansion ☐ Depletion ☐ Activation / state change ☐ Immune reconstitution ☐ Compartment-restricted finding ☐ Product dose / quality ☐ Other: ___________________________________________________ | | | | | | | |
| Relative vs absolute discordance: ☐ No ☐ Yes | | | | Context-congruent finding: ☐ Yes ☐ No ☐ Uncertain | | | |
| Compartment specificity materially affects meaning: ☐ No ☐ Yes | | | | Need for longitudinal comparison: ☐ No ☐ Yes | | | |
| Aberrant Phenotype Detection: ☐ No ☐ Yes | | | |  | | | |
| Integrated interpretive rationale  _______________________________________________________________________________________________  _______________________________________________________________________________________________  _______________________________________________________________________________________________ | | | | | | | |
| Recommended reporting action  ☐ Clinical correlation ☐ Repeat under same conditions ☐ Longitudinal follow-up ☐ Compare with prior result ☐ Complementary assay ☐ Urgent specialist review ☐ Clinical Urgency Flag | | | | | | | |
| **7. Final report statement** | | | | | | | |
| Final interpretive impression  _______________________________________________________________________________________________  _______________________________________________________________________________________________  _______________________________________________________________________________________________  _______________________________________________________________________________________________ | | | | | | | |
| Name of the analysis software and version: _____________________________________________________ | | | | Platform / Instrument:: ___________________________________ | | | |
| Reported by: ____________________ | | | | Signature / date: __________________ | | | |
